# Regulatory effects of transcription factor PAX3 on human dental pulp stem cells

**DOI:** 10.1186/s12903-026-09117-5

**Published:** 2026-07-07

**Authors:** Zengguo Chen, Huijuan Liu, Xue Zhang, Dandan Li, Wenjing Li

**Affiliations:** 1 Department of Oral Medicine, The Second Hospital of Hebei Medical University, Shijiazhuang, 050000 China; 2 Hebei Key Laboratory of Stomatology, Hebei Technology Innovation Center of Oral Health, School and Hospital of Stomatology, Hebei Medical University, Shijiazhuang, 050017 China; 3 Department of Cariology and Endodontics, Jinan Stomatological Hospital, Jinan, 250001 China

**Keywords:** PAX3, Dental pulp stem cells, Senescence, Proliferation, Osteogenic differentiation

## Abstract

**Background:**

Dental pulp stem cells (DPSCs) are promising for regenerative dentistry but undergo replicative senescence during in vitro expansion, leading to diminished proliferative capacity and osteogenic potential. This study aimed to investigate the role of the transcription factor PAX3 in regulating the senescence process of human DPSCs (hDPSCs).

**Methods:**

A replicative senescence model of hDPSCs was established through serial passaging (P3 as young cells vs. P11 as senescent cells). The expression of PAX3 and senescence markers (p53, p21) was assessed. The functional role of PAX3 was investigated by knocking it down (si-PAX3) or overexpressing it (OE-PAX3) in hDPSCs, followed by evaluations of senescence-associated β-galactosidase (SA-β-gal) activity, cell proliferation, and osteogenic differentiation.

**Results:**

PAX3 expression was significantly downregulated in senescent P11 hDPSCs compared to young P3 cells. Functionally, PAX3 knockdown was associated with accelerated cellular senescence, as evidenced by increased SA-β-gal activity, upregulation of p53 and p21 mRNA levels, impaired cell proliferation, and reduced osteogenic differentiation. Conversely, PAX3 overexpression was associated with an attenuated senescent phenotype, resulting in decreased SA-β-gal activity and expression of p53/p21, enhanced proliferation, and improved osteogenic capacity.

**Conclusion:**

The transcription factor PAX3 may play a critical regulatory role in mitigating hDPSCs senescence, as downregulation of PAX3 was associated with increased senescence markers and its overexpression with reduced senescence markers. The observed correlation with p53/p21 mRNA levels suggests that this pathway may be involved, but mechanistic validation at the protein level is still required. These findings indicate that PAX3 represents a candidate for further investigation as a potential target to delay senescence and maintain the functional properties of hDPSCs, warranting further investigation for its potential utility in tissue engineering and clinical stem cell-based therapies.

## Introduction

 Aging is a progressive process characterized by functional decline and disruption of homeostasis over time. Previous studies have shown that during age-related changes in dental pulp tissue, progressive degeneration occurs. In elderly individuals, the dental pulp stem cell (DPSC) population exhibits reduced cell density, impaired tissue repair capacity, decreased metabolic activity, and imbalance in redox homeostasis. In addition, senescence attenuates the proliferative and differentiation potential of DPSCs [1–3]. Clinically, the success of vital pulp preservation and pulp regeneration therapies relies on the self-renewal, proliferation, and differentiation capacity of DPSCs; therefore, the efficacy of these treatments diminishes with pulp aging. Since the number of DPSCs that can be isolated and cultured from dental pulp is limited, large-scale expansion is required to meet the demands of clinical and tissue engineering applications. In vitro expansion is typically achieved through continuous passaging; however, previous studies have demonstrated that serially passaged DPSCs also undergo senescence, characterized by DNA damage, growth arrest, and increased β-galactosidase activity [4, 5]. Senescent DPSCs display markedly reduced proliferative capacity and loss of differentiation potential, ultimately resulting in insufficient cell numbers for research and clinical use. Thus, preserving the proliferative and differentiation potential of DPSCs during in vitro expansion is a critical prerequisite for their scientific and therapeutic applications.

Paired box gene 3 (PAX3) is a member of the PAX family of transcription factors, located at chromosome 2q36.1. It is characterized by a highly conserved paired-box domain and functions as a key regulator of mammalian growth and development. During embryogenesis, PAX3 plays an essential role in skeletal myogenesis, neural crest cell migration and differentiation, and central nervous system morphogenesis, where it regulates the expression of target genes involved in proliferation, survival, differentiation, and motility [6, 7]. Furthermore, studies have shown that PAX3 plays a crucial role in the proliferation, invasion, and apoptosis of cancer cells, including gastric cancer [8–10], prostate cancer [11], and melanoma [12]. Based on these findings, we hypothesized that PAX3 may also participate in regulating the proliferation and differentiation of DPSCs and may be involved in the control of DPSC senescence. However, the role of PAX3 in the senescence of DPSCs has not yet been reported.

In this study, human DPSCs (hDPSCs) were cultured in vitro, and a cellular senescence model was established through serial passaging. The expression of PAX3 in young and senescent hDPSCs was compared, and the role of PAX3 in regulating hDPSC senescence was investigated. The aim was to address the technical bottleneck posed by replicative senescence during in vitro expansion that limits the clinical translation of hDPSCs, and to provide new insights and theoretical basis for their application in tissue engineering and stem cell–based therapies.

## Materials and methods

The study period was from June 2023 to January 2025. The research plan and the acquisition of human ex vivo teeth were both approved by the Ethics Committee of the Second Hospital of Hebei Medical University (Approval Number: 2022-R-248). All procedures involving human participants were conducted in accordance with the Declaration of Helsinki. Informed consent was obtained from all individual donors whose teeth were used in this study.

### Cell culture and identification of hDPSCs

The human dental pulp stem cells used in this study were isolated from dental pulp tissues obtained from the Biobank of The Second Hospital of Hebei Medical University. hDPSCs were derived from independent donors, with a donor age range of 18–25 years. The original tissue samples were collected from extracted teeth with prior donor consent for research use. All experiments were repeated at least three times using independently isolated cell batches from different donors to account for inter-donor variability.

Human dental pulp stem cells were primary cultured by the tissue block method. Cells were cultured in α-minimum essential medium (α-MEM, Gibco, USA) supplemented with 10% fetal bovine serum (FBS, Gibco, USA), 100 U/mL penicillin, and 100 µg/mL streptomycin at 37 °C in a humidified atmosphere containing 5% CO₂. The culture medium was changed every 3 days. The dental pulp stem cells were identified by HE staining, immunohistochemical staining and flow cytometry. For flow cytometry analysis, hDPSCs at P3 were detached and incubated with the following fluorescein isothiocyanate (FITC)- or phycoerythrin (PE)-conjugated monoclonal antibodies: anti-CD44-FITC (Clone: G44-26, BD Biosciences, USA), anti-CD90-PE (Clone: 5E10, BD Biosciences, USA), anti-CD34-PE (Clone 581, BD Biosciences, USA), and anti-CD45-FITC (Clone HI30, BD Biosciences, USA). Corresponding isotype-matched mouse IgG1-FITC, IgG1-PE, and IgG2a-PE (BD Biosciences, USA) were used as negative controls to establish background fluorescence and gate settings. Cells were analyzed using a FACSCalibur flow cytometer (BD Biosciences, USA), and data were processed with FlowJo software (version 10.0, TreeStar, USA). A minimum of 10,000 events were acquired per sample.

Experimental grouping: Group I: According to different generations of hDPSCs, it was divided into the young cell group (Generation 3, P3) and the old cell group (Generation 11, P11). Group II: The silencing of PAX3 by hDPSCs was divided into the silencing group (si-PAX3) and the control group 1 (si-NC). Group III: hDPSCs overexpressing PAX3 were divided into the overexpression group (OE-PAX3) and the empty vector control group 2 (OE-NC).

### Cell transfection

siRNA sequences targeting PAX3 and negative control siRNA were designed and synthesized by Hebei Benyuan Biotechnology Co., Ltd. (Shijiazhuang, China). For PAX3 overexpression, the full-length human PAX3 coding sequence was amplified and cloned into the pcDNA3.1(+) vector (Invitrogen, USA). The empty pcDNA3.1(+) vector was used as the negative control (OE-NC). For transfection, P3 hDPSCs were seeded in 6-well plates and cultured until they reached 70% confluence. Transfection was performed using UltraFection 3.0 reagent (4 A Biotech, China) according to the manufacturer’s instructions. 48 h after transfection, transfection efficiency was evaluated by fluorescence microscopy (Olympus, Japan) and confirmed by real-time fluorescence quantitative PCR (RT-qPCR).

### RNA extraction and quantitative real-time PCR

Total RNA was extracted from hDPSCs using TRIzol reagent (Invitrogen, USA) according to the manufacturer’s protocol. RNA concentration and purity were assessed using a NanoDrop spectrophotometer (Thermo Fisher Scientific, USA). Reverse transcription was performed using a PrimeScript RT Reagent Kit (Takara, Japan). Real-time PCR was carried out using SYBR Green Master Mix (Takara, Japan) on a LightCycler 480 system (Roche, Switzerland). Gene-specific primers were designed using Primer Premier 5.0 (Table 1). The *GAPDH* gene was chosen as the endogenous control, with relative gene expression levels quantified via the 2^−△△Ct^ method [13].

**Table 1:** Sequences of primers used for real-time PCR

Target gene	Accession No.	Primer sequences (from 5’ to 3’)
*p53*	NM_000546	F: CCTCAGCATCTTATCCGAGTGG
		R: TGGATGGTGGTACAGTCAGAGC
*p21*	NM_000389	F: AGGTGGACCTGGAGACTCTCAG
		R: TCCTCTTGGAGAAGATCAGCCG
PAX3	NM_181459	F: GGCTTTCAACCATCTCATTCCCG
		R: GTTGAGGTCTGTGAACGGTGCT
*GAPDH*	NM_002046	F: GTCTCCTCTGACTTCAACAGCG
		R: ACCACCCTGTTGCTGTAGCCAA

### Cell proliferation assay

Cell proliferation was assessed using the Cell Counting Kit-8 (CCK-8, Dojindo, Japan). hDPSCs from each experimental group were trypsinized, counted, and seeded into 96-well plates at a density of 2 × 10³ cells per well with 5 replicate wells per group. At days 1, 3, 5, and 7, CCK-8 reagent (10 µL per well) was added and incubated for 2 h at 37 °C. The absorbance at 450 nm was measured using a microplate reader (Bio-Rad, USA), and cell growth curves were plotted.

### Osteogenic differentiation assay

To evaluate osteogenic differentiation potential, hDPSCs were seeded into 6-well plates at a density of 3 × 10⁵ cells per well and cultured in osteogenic induction medium (Cyagen Biosciences, China). After 7 days of induction, alkaline phosphatase (ALP) staining was performed using a BCIP/NBT ALP color development kit (Beyotime, China). After 21 days of induction, alizarin red staining was performed to visualize mineralized nodules. Staining results were observed and photographed under an inverted microscope (Olympus, Japan). Mineralized nodule formation was assessed by semi-quantitative counting of alizarin red-positive nodules in five random fields per well.

### Senescence-associated β-galactosidase (SA-β-Gal) staining

Cellular senescence was assessed using a senescence-associated β-galactosidase staining kit (Beyotime, China). hDPSCs were seeded into 6-well plates at a density of 3 × 10⁵ cells per well. After culture, cells were fixed and stained according to the manufacturer’s protocol. Stained cells were incubated at 37 °C for 24 h, and blue-green stained cells were observed and counted under an optical microscope (Olympus, Japan). The percentage of SA-β-gal-positive cells was calculated from at least five random fields per well.

### Statistical analysis

All experiments were performed at least three times independently. Statistical analysis was performed using GraphPad Prism 10.0 software (GraphPad Software, USA). Data are presented as mean ± standard deviation (SD). Normality and homogeneity of variance were confirmed using the Shapiro-Wilk test and Levene’s test, respectively. An independent samples t-test was used for comparisons between two groups. One-way analysis of variance (ANOVA) followed by the least significant difference (LSD) post hoc test was used for comparisons among multiple groups. A two-sided *P* value < 0.05 was considered statistically significant.

## Result

### Primary culture of dental pulp stem cells

**Fig. 1:**
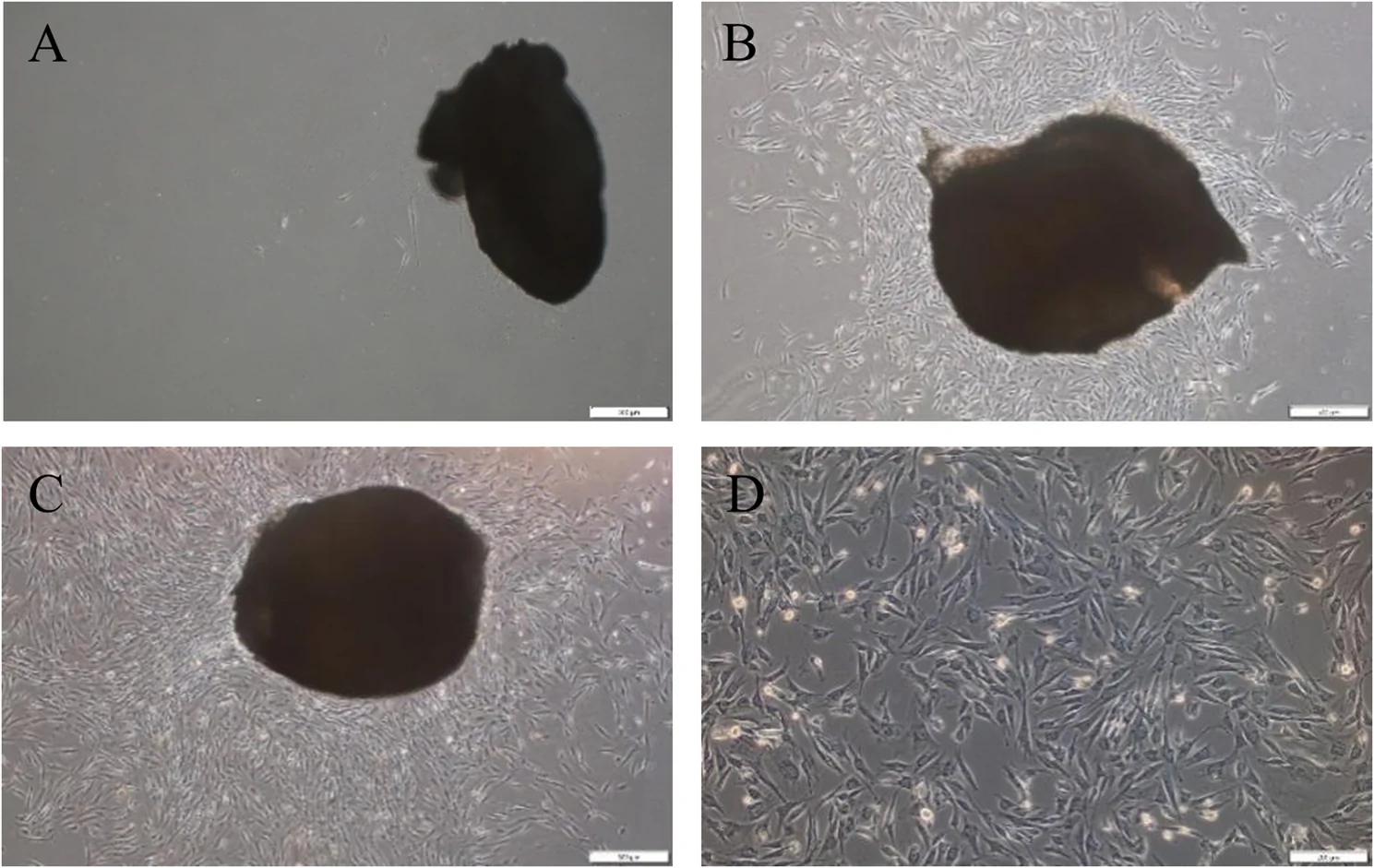
Primary culture of hDPSCs. **A** After 7 days of culture, hDPSCs crawled out from around the tissue block (40×); **B** After 15 days of culture, hDPSCs expanded around the tissue block (40×); **C** After 20 days of culture, hDPSCs reached the passage density (40×); **D** hDPSCs grew in a vortex pattern after passage (100×). Scale bar = 500 μm for (**A**–**C**), and 200 μm for (**D**)

Dental pulp stem cells can be successfully cultured by the tissue block adherent method. After 5 to 14 days of culture, short spindle cells can be observed to climb out from around the tissue block (Fig. 1A). When the culture continues, we can see that the cell density around the tissue block gradually increases and continuously expands outward, and radiates in a concentric circular pattern centered on the tissue block (Fig. 1B). The first passage is carried out when the cell density reaches 80% (Fig. 1C). After passage, the cells can be observed to grow in a vortex pattern (Fig. 1D). The second passage can be conducted when the cell density reaches 80%.

### Identification of human dental pulp stem cells

**Fig. 2:**
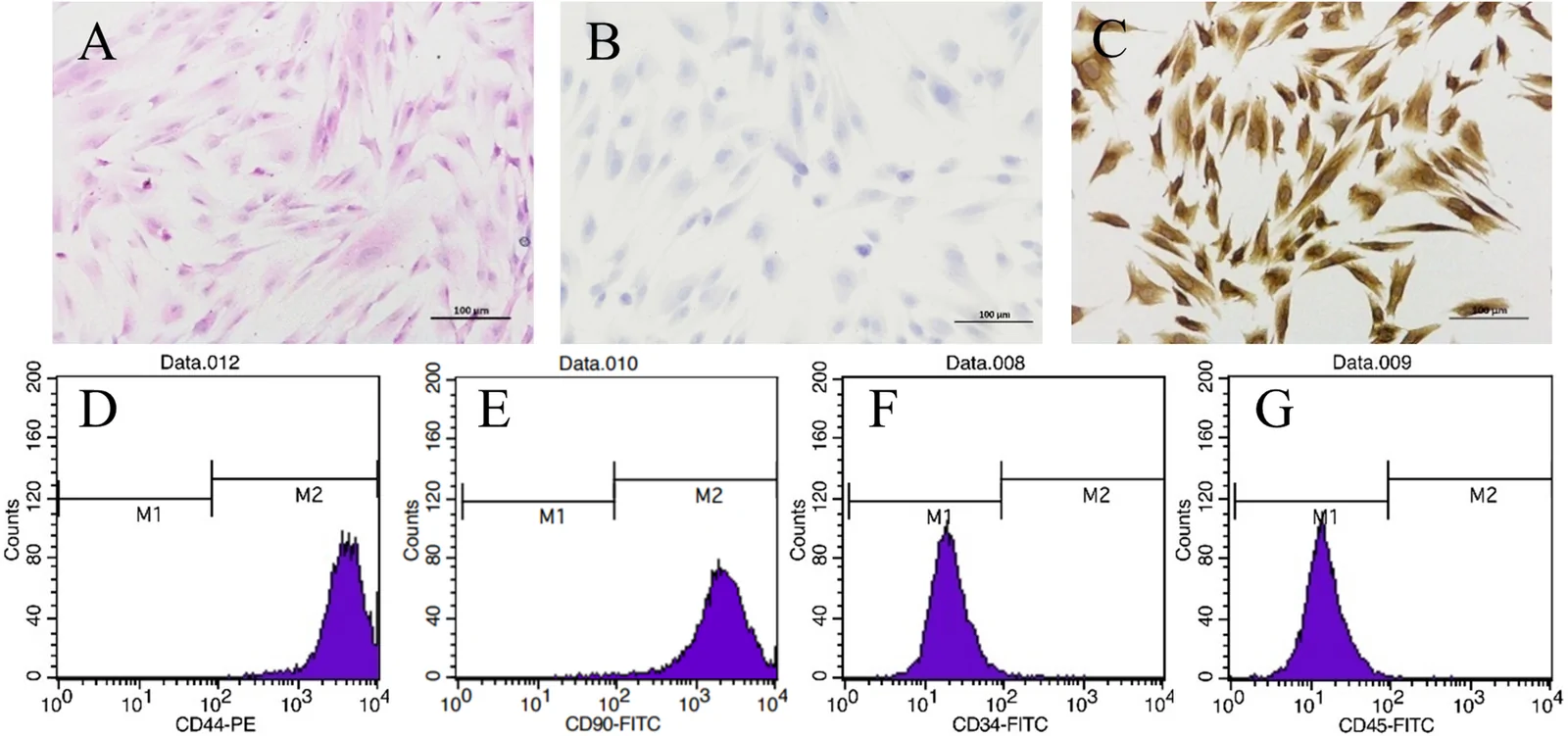
Identification of hDPSCs. **A** HE staining (200×); **B** Keratin staining negative (200×); **C** Vimentin staining positive (200×); **D**-**G** Identification of surface markers of hDPSCs by flow cytometry. Scale bar = 100 μm

HE staining shows that the cells are distributed in bundles, the cell bodies are long spindle-shaped, the cell nuclei are located in the center of the cells and are round or oval, the cytoplasm is eosinophilic, and the cell nuclei are basophilic (Fig. 2A). Immunohistochemical staining showed that CK staining was negative (Fig. 2B) and VIM staining was positive (Fig. 2C). The flow cytometry results showed that CD44 (98.7%) and CD90 (96.65%) were highly expressed in the extracted cells, while CD34 (0.6%) and CD45 (0.2%) showed lower expression in the extracted cells (Fig. 2D-G). These results have fully demonstrated that the cells extracted by the tissue block adherent method in this study are hDPSCs.

### Comparison of cell viability and morphology before and after continuous passage

**Fig. 3:**
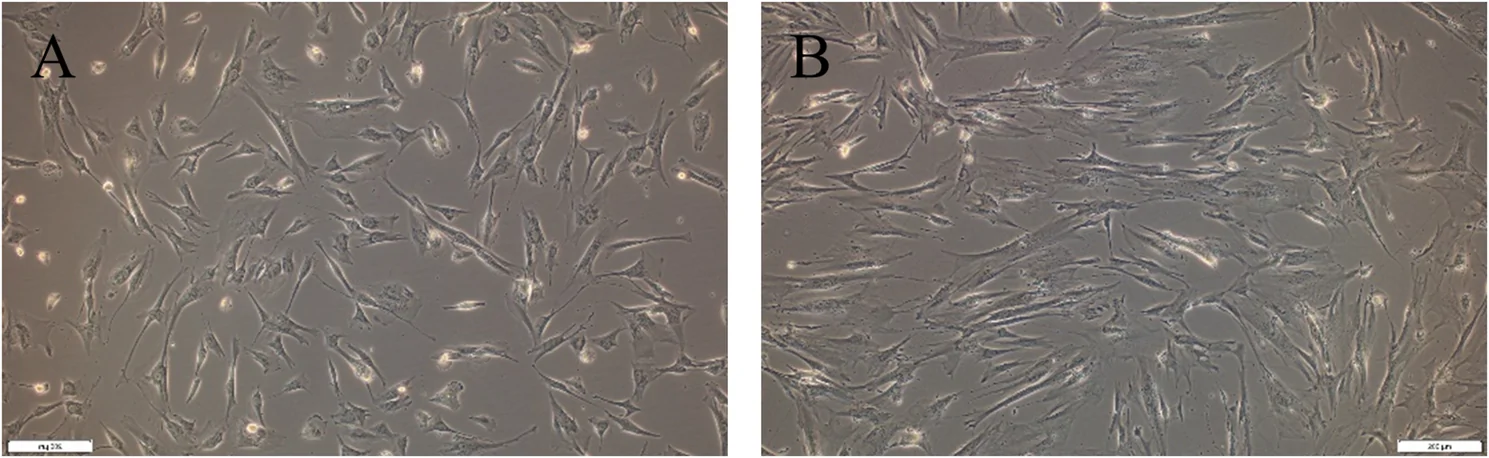
Primary culture of hDPSCs. **A** P3 hDPSCs (100×); **B** P11 hDPSCs (100×). Scale bar = 200 μm

The growth state of P11 dental pulp stem cells (Fig. 3B) was worse than that of P3 dental pulp stem cells (Fig. 3A), with increased volume, flattened shape and increased length.

### Observation of transfection effect

**Fig. 4:**
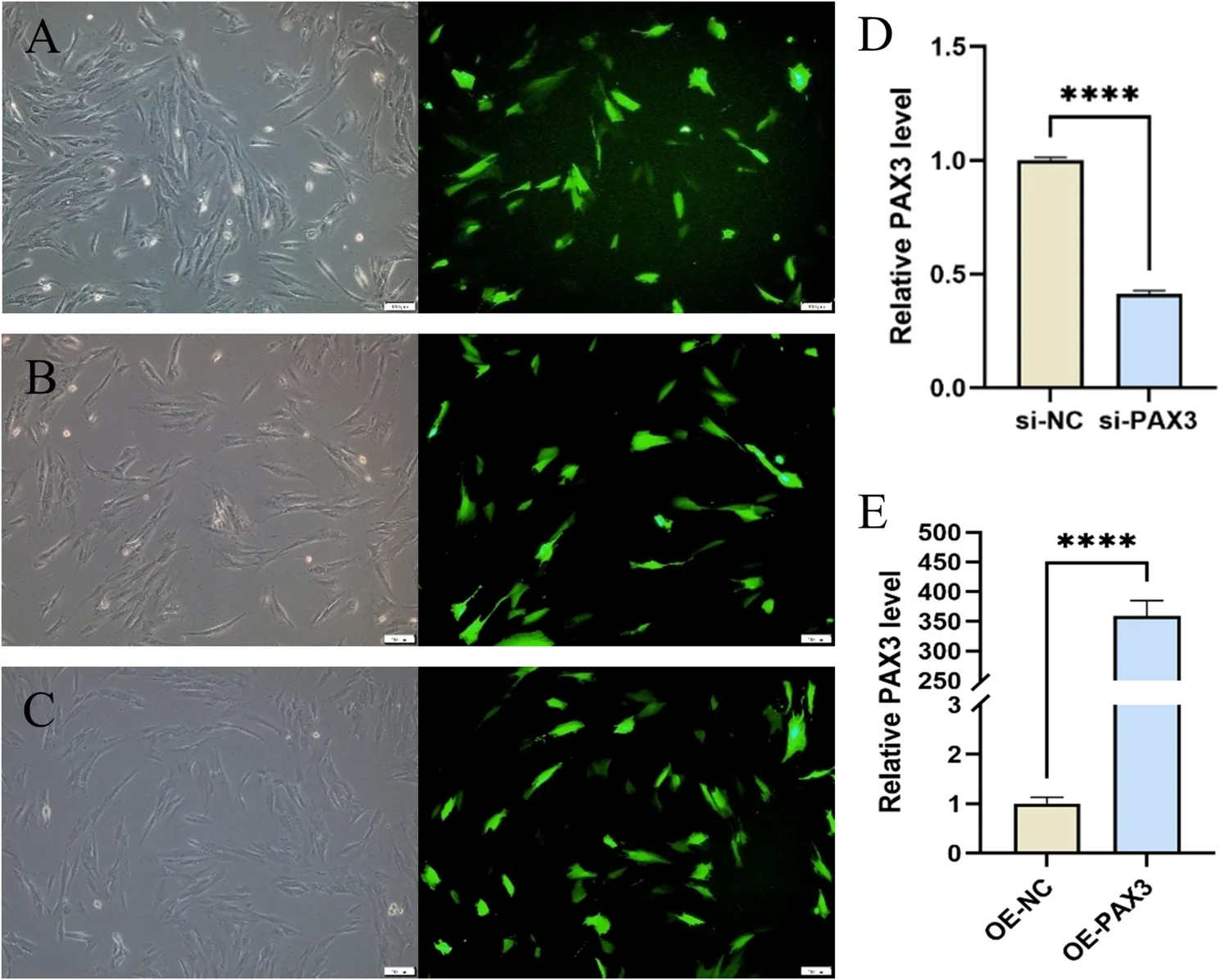
Verification of transfection efficiency. **A** Fluorescence expression of si-FAM 48 h after transfection (100×); **B** Fluorescence expression of OE-PAX3 48 h after transfection (100×); **C** Fluorescence expression of OE-NC 48 h after transfection (100×); **D** The silencing efficiency of si-PAX3 was verified at the mRNA level 48 h after transfection; **E** Verify the overexpression efficiency of OE-PAX3 at the mRNA level 48 h after transfection. Scale bar = 100 μm

Green fluorescence was observed in the cells of both Group II and Group III hDPSC 48 h after transfection under a fluorescence microscope (Fig. 4).

### RT-qPCR detection results

**Fig. 5:**
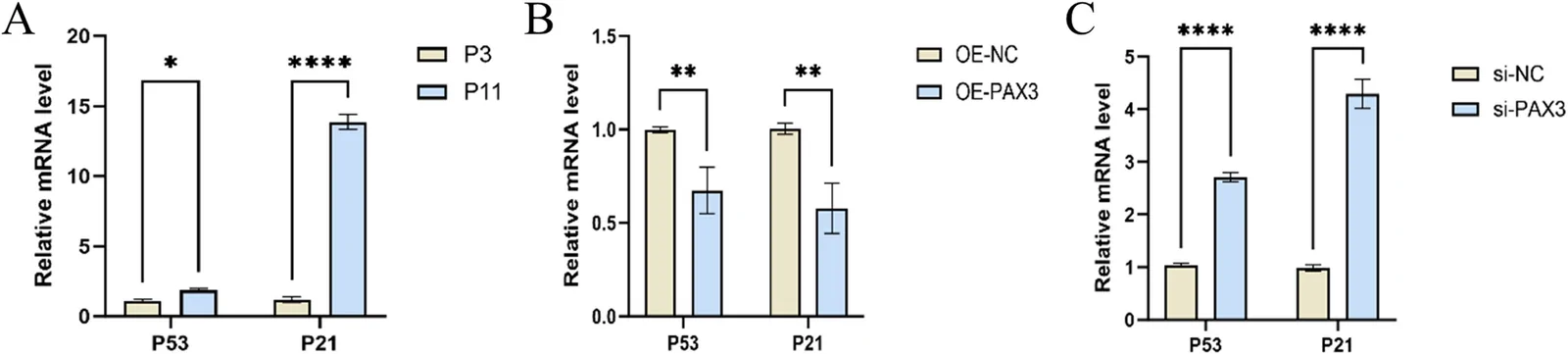
RT-qPCR verification shows the changes in the expression levels of P53 and P21 in (**A**) group I, (**B**) group II, and (**C**) group III

In group I, compared with P3, the expression level of p53 and p21 in P11 hDPSCs was significantly upregulated (*P < 0.05 **P < 0.01) (Fig. 5A). In groups II and III, the expressions of p53 and p21 at the mRNA level in the OE-PAX3 group were significantly downregulated compared with the OE-NC group (**P < 0.01 **P < 0.01) (Fig. 5B). The expression levels of p53 and p21 in the si-PAX3 group were upregulated compared with those in the si-NC group (****P < 0.0001, ****P < 0.0001) (Fig. 5C).

### CCK-8 assay results

**Fig. 6:**
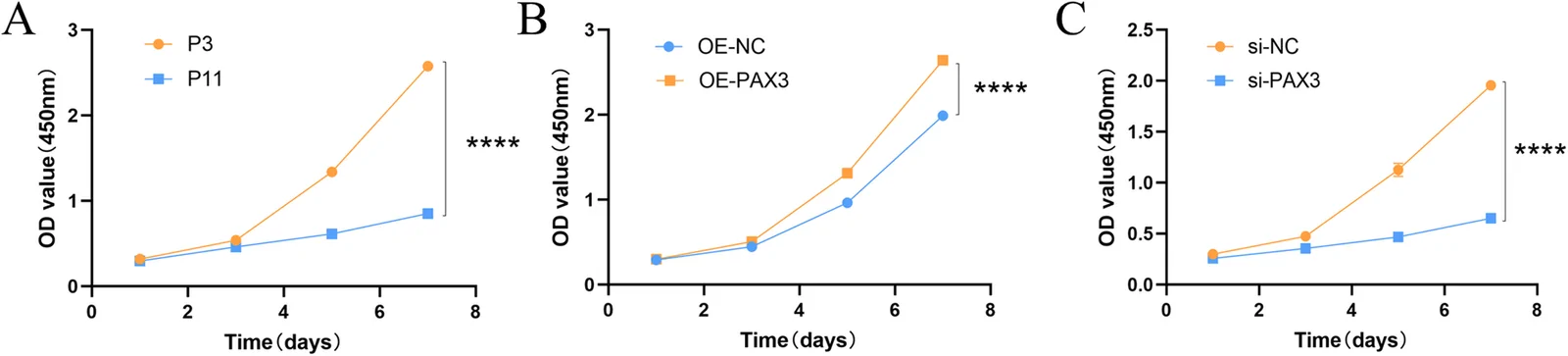
CCK-8 verification of cell proliferation rate. **A** Group I; **B** Group II; C Group III

The OD values of both P3 and P11 hDPSCs showed an upward trend, but the OD value of P3 increased faster and was higher than that of P11 (****P < 0.0001) (Fig. 6A). The OD values of the OE-PAX3 group and the OE-NC group both showed an upward trend. The OD value of the OE-PAX3 group increased faster and was always higher than that of the OE-NC group (****P < 0.0001) (Fig. 6B). The OD values of both the si-PAX3 group and the si-NC group showed an upward trend, while the OD value of the si-PAX3 group increased more slowly and consistently was lower than that of the si-NC group (****P < 0.0001) (Fig. 6C).

### Osteogenic induction results

**Fig. 7:**
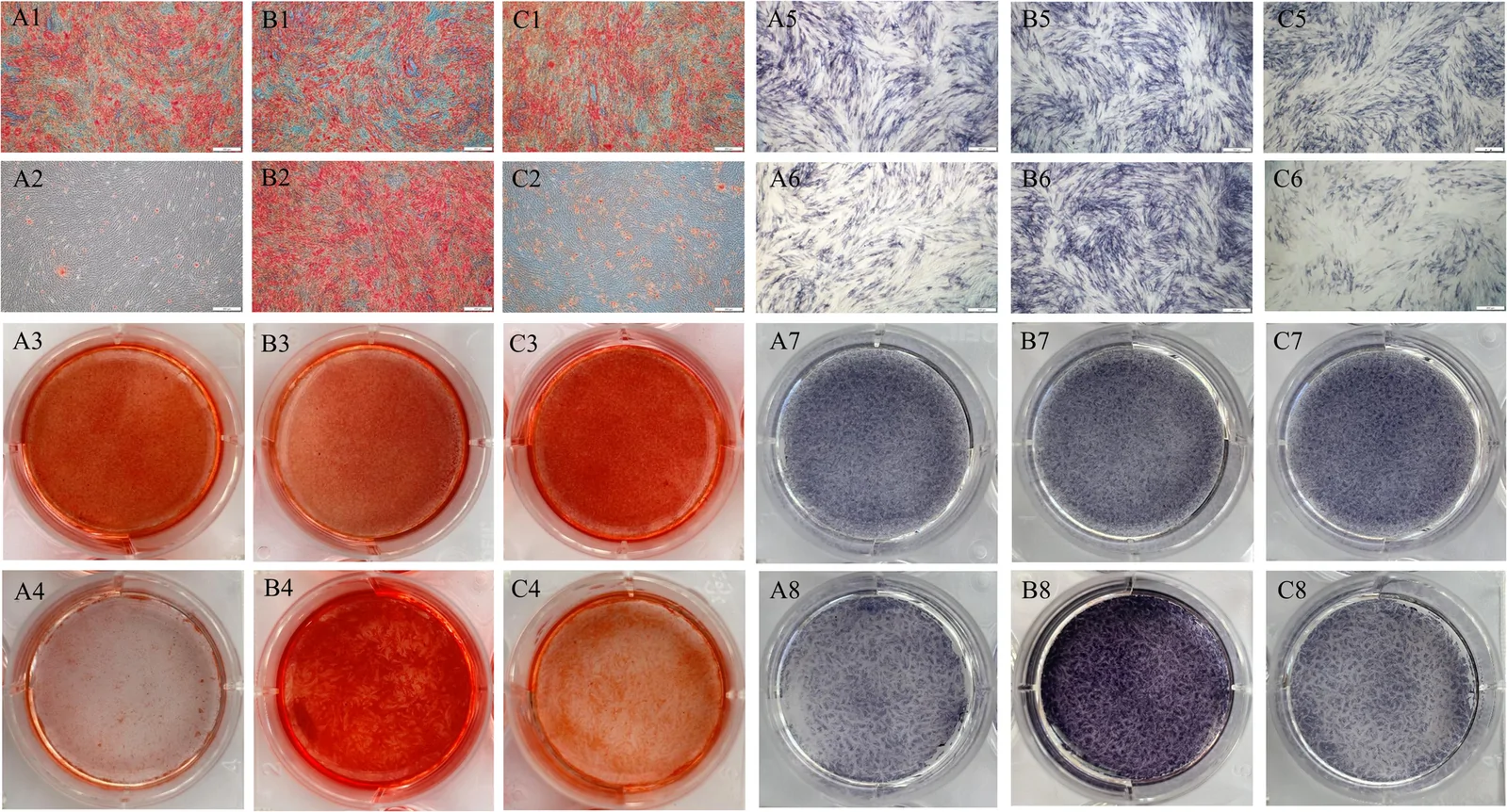
Representative images of osteogenic differentiation assessment. Scale bar = 200 μm. **A1** P3 under alizarin red microscope; **A2** P11 under alizarin red staining microscope; **A3** P3 under alizarin red staining intuitive view; **A4** P11 under Alizarin red staining intuitive view; **A5** P3 under ALP staining microscope; **A6** P11 under ALP staining microscope; **A7** P3 under ALP staining intuitive view; **A8 **P11 ALP staining intuitive view. **B1** Observation under OE-NC alizarin red microscope; **B2** Observation under OE-PAX3 alizarin red staining microscope; **B3** Intuitive chart of OE-NC alizarin red staining; **B4** Intuitive chart of OE-PAX3 alizarin red staining; **B5** Observation under OE-NC ALP staining microscope; **B6** Observation under OE-PAX3 ALP staining microscope; **B7** Observation chart of OE-NC ALP staining; **B8** Observation chart of OE-PAX3 ALP staining. **C1** Observation under the microscope of si-NC alizarin red; **C2** Observation under the microscope of si-PAX3 alizarin red staining; **C3** Intuitive chart of si-NC alizarin red staining; **C4** Intuitive chart of si-PAX3 alizarin red staining; **C5** Observation under the microscope of si-NC ALP staining; **C6** Observation under the microscope of si-PAX3 ALP staining; **C7** Direct view of si-NC ALP staining; **C8** Direct view of si-PAX3 ALP staining

In group I, the blue-purple staining of P3 hDPSCs was darker than that of P11, suggesting a higher activity of alkaline phosphatase. The orange-red staining of P3 hDPSCs is deeper than that of P11. Under an inverted microscope, it can be observed that the cells accumulate in a vortex pattern, with mineralized nodules formed and deeply stained orange-red. There are more and denser orange-red mineralized nodules in P3 hDPSCs than in P11 (Fig. 7A1-A8). The semi-quantitative analysis results showed that the formation numbers of mineralized nodules in P3 and P11 were (20.67 ± 0.47) per hole and (4.33 ± 0.94) per hole, respectively. In Group II, the OE-PAX3 group was stained deeper than the OE-NC group, suggesting a higher activity of alkaline phosphatase. The OE-PAX3 group was stained deeper than the OE-NC group. Under the inverted microscope, it was observed that there were more mineralized nodules in the OE-PAX3 group than in the OE-NC group (Figs. 7B1-B8). The semi-quantitative analysis results showed that the number of mineralized nodules formed in the OE-NC group and the OE-PAX3 group was (21.33 ± 0.51) nodules/hole and (30.22 ± 0.94) nodules/hole, respectively. In Group III, the staining of the si-PAX3 group was lighter than that of the si-NC group, suggesting lower activity of alkaline phosphatase. The staining of the si-PAX3 group was lighter than that of the si-NC group. Under the inverted microscope observation, it could be seen that the mineralized nodules in the si-PAX3 group were reduced (Fig. 7C1-C8) compared with the si-NC group. The semi-quantitative analysis results showed that the number of mineralized nodules formed in the si-NC group and the si-PAX3 group was (20.33 ± 0.41) nodules/hole and (7.22 ± 0.94) nodules/hole, respectively.

### β -galactosidase (SA-β -GAL) staining results

**Fig. 8:**
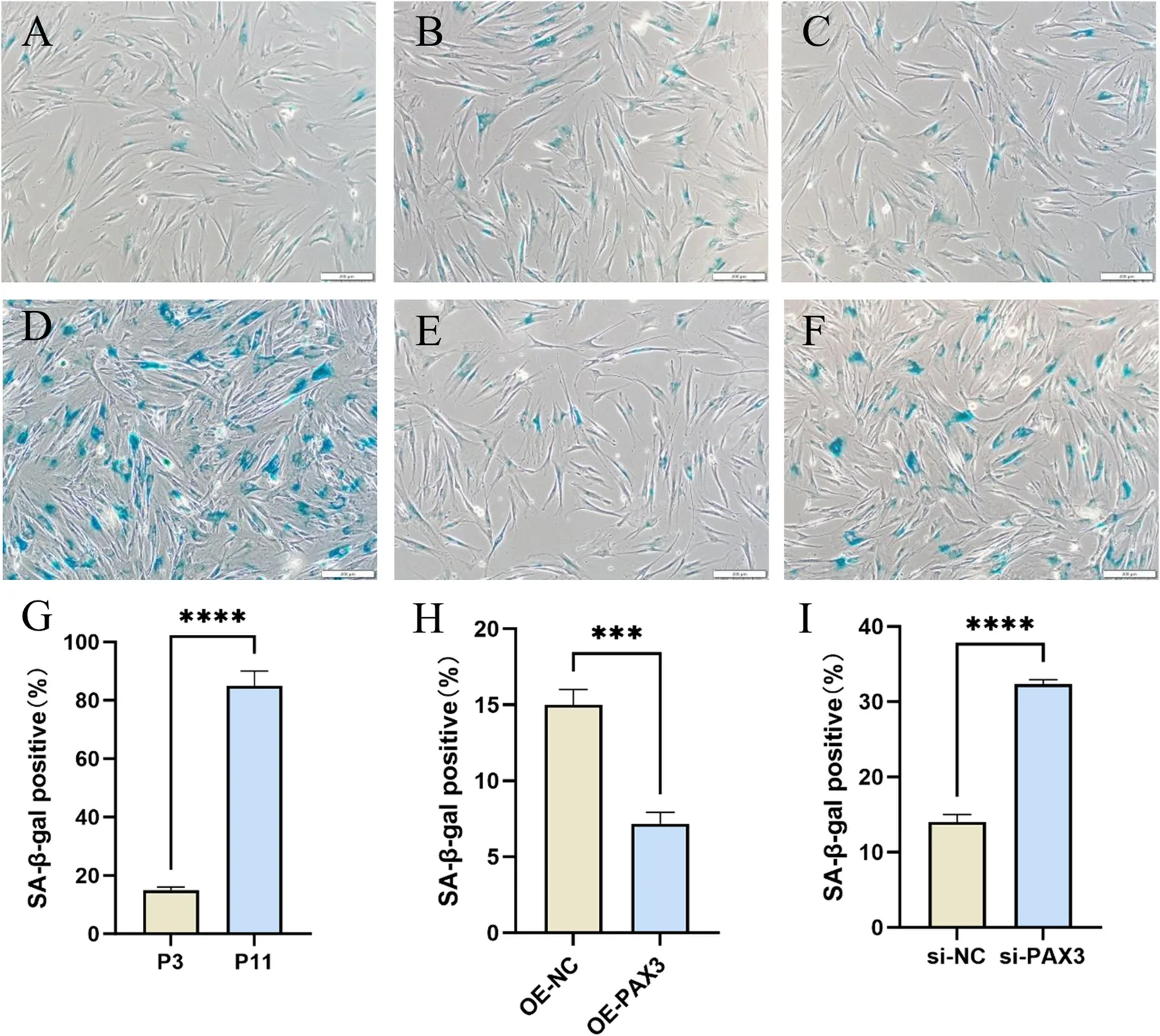
SA-β-GAL staining for verifying the positive rate of cell senescence. Scale bar = 200 μm. **A** microscopic observation of P3-generation hDPSCs SA-β-GAL staining (100×); **B** microscopic observation of OE-NC hDPSCs SA-β-GAL staining (100×); **C** microscopic observation of si-NC hDPSCs SA-β-GAL staining (100×); **D** microscopic observation of P11-generation hDPSCs SA-β-GAL staining (100×); **E** microscopic observation of OE-PAX3 hDPSCs SA-β-GAL staining (100×); **F** microscopic observation of si-PAX3 hDPSCs SA-β-GAL staining (100×); **G** comparison of the positive rate of SA-β-GAL staining in group I; **H** comparison of the positive rate of SA-β-GAL staining in group II; **I** comparison of the positive rate of SA-β-GAL staining in group III

In Groups I, II, and III, the positive rates of senescence staining were increased in the P11, OE-NC, and si-PAX3 groups compared with the P3, OE-PAX3, and si-NC groups, respectively. (*P* < 0.05) (Fig. 8).

## Discussion

Teeth are critical masticatory organs in humans, and the dental pulp— the sole soft tissue within dental structures—plays an irreplaceable role in supporting physiological functions such as mastication and sensory perception. Dental pulp exhibits limited resistance to external stimuli; clinically, it is frequently exposed or infected due to factors like dental caries, periodontitis, and trauma, often leading to irreversible pulp necrosis. Root canal therapy remains the primary treatment for pulp necrosis, but it inflicts irreversible damage to the tooth: post-treatment, the tooth becomes less resistant to occlusal forces, increasing susceptibility to fracture. Moreover, the survival rate of treated teeth has been reported to decline progressively over time and many surviving teeth may develop complications such as periodontitis [14]. Given these limitations, regenerative pulp therapy has emerged as a promising alternative. However, a major challenge in this field is stem cell senescence, which significantly impairs the osteogenic and proliferative capacities of DPSCs, thereby compromising regenerative efficacy. Thus, investigating the mechanisms underlying DPSCs senescence and developing targeted interventions holds substantial significance for advancing tissue engineering and regenerative medicine.

Aging is a time-dependent process characterized by progressive functional decline and disrupted homeostasis, serving as a common risk factor for degenerative diseases including Alzheimer’s disease [15], cardiovascular diseases [16], and osteoarthritis [17]. It can also impair the body’s ability to recognize cancerous cells and induce systemic organ degeneration, rendering the elderly particularly vulnerable to chronic diseases. A core hallmark of aging is the decline in tissue repair capacity and immune surveillance, which is mirrored in dental pulp tissue: age-related changes include reduced volume, diminished cellular activity, and eventual loss of function in the pulpodentin complex [18], accompanied by tooth discoloration (yellowing/blackening), brittleness, and increased susceptibility to dental pathologies, tissue defects, or premature tooth loss.

Stem cell senescence is a complex biological process, characterized by downregulated expression of stem cell markers, impaired proliferation, skewed differentiation potential, and a deteriorated microenvironment. Its molecular mechanisms involve mitochondrial dysfunction, oxidative stress, protein homeostasis imbalance, genomic instability, accumulated DNA damage, telomere attrition, and epigenetic alterations [19]. Human dental pulp stem cells (hDPSCs) possess robust differentiation potential and regenerative capacity, making them ideal seed cells for dental tissue regeneration. While hDPSCs can be isolated and cultured from dental pulp, large quantities are required for research and clinical applications, necessitating in vitro expansion. However, under standard culture conditions, cells undergo replicative senescence due to limited division potential, hindering experimental progress and clinical translation. Additionally, age-related changes in dental pulp tissue result in more pronounced senescence in hDPSCs isolated from older donors compared to younger counterparts. Relative to young hDPSCs, senescent hDPSCs exhibit reduced self-renewal, migration, and osteogenic differentiation, weakening dental healing and regeneration— a critical limitation in tissue engineering and stem cell-based therapies.

In this study, we cultured hDPSCs in vitro and confirmed their identity as mesenchymal stem cells (MSCs) via surface antigen detection and immunohistochemical staining. To establish a replicative senescence model, we induced hDPSCs senescence via continuous passaging and observed morphological changes across generations. Microscopic analysis of multiple specimens revealed that by passage 11 (P11), hDPSCs exhibited significantly larger volume, elongated morphology, and flattened appearance compared to passage 3 (P3) cells. These morphological changes are consistent with previous reports in other MSC types [20, 21]. Thus, P3 and P11 hDPSCs were selected as control and senescent models, respectively, for subsequent experiments.

To further validate the senescent phenotype, we assessed senescence-associated β-galactosidase (SA-β-gal) activity—a classic senescence marker—which catalyzes the formation of a deep blue product with the substrate X-gal (5-bromo-4-chloro-3-indolyl-β-D-galactoside) at pH 6.0 [22]. SA-β-gal staining in our study showed a higher positive rate in P11 hDPSCs compared to P3 cells, confirming the senescent phenotype of P11 cells.

Similarly, p53 and p21, well-recognized senescence-associated markers, were significantly upregulated at the mRNA level in P11 hDPSCs compared to P3 cells. These findings collectively validate that P11 hDPSCs represent a reliable model of replicative senescence. The transcription factor p53 is primarily known for its tumor-suppressive functions, regulating cancer cell proliferation, senescence, DNA repair, and death through transcriptional and non-transcriptional activities [23]. The cyclin-dependent kinase inhibitor p21 (also referred to as WAF1, CIP1, SDI1, or MDA-6) induces G1-phase arrest, preventing cells from entering S phase; its overexpression effectively inhibits tumor growth in vitro and in vivo [24]. In our study, mRNA levels of p53 and p21 were significantly higher in P11 hDPSCs than in P3 cells, confirming the senescent status of P11 hDPSCs.

In regenerative medicine, hDPSCs are valued for their applications in pulp regeneration, neural injury repair, and bone defect regeneration (due to their inducible osteogenic potential). Studies have shown that DPSCs combined with various scaffolds can enhance osteogenic differentiation, supporting their use in treating bone defects [25–27]. Given that hDPSCs status directly influences osteogenic capacity, we assessed in vitro osteogenic potential using osteogenic induction medium, finding that P11 hDPSCs exhibited significantly reduced osteogenic ability compared to P3 cells.

PAX3 belongs to the PAX family of transcription factors, which comprises 9 members (PAX1-PAX9) that bind specific DNA sequences to regulate target gene expression. PAX3 is a key regulator of embryonic myogenesis, promoting mesenchymal stem cell (MSC) differentiation into myocytes [28, 29]. PAX3 also plays critical roles in cancer cell proliferation, invasion, and apoptosis. For example, silencing PAX3 impairs gastric cancer cell proliferation, promotes apoptosis, and inhibits angiogenesis by upregulating pro-apoptotic caspase-3, downregulating vascular endothelial growth factor (VEGF), and suppressing the MET/PI3K pathway [8]. Similarly, miR-299-3p and miR-206 inhibit gastric cancer progression by downregulating PAX3, with miR-206 deficiency leading to PAX3 overexpression and MET-mediated metastasis [9, 10]. In prostate cancer, PAX3 silencing inhibits proliferation and metastasis by suppressing the TGF-β/Smad pathway [11], while bitter melon extract inhibits melanoma progression by downregulating PAX3 and its downstream PI3K/Akt/mTORC and MITF pathways [12]. In glioma stem cells (GSCs), PAX3 silencing reduces proliferation and invasion while promoting apoptosis, whereas overexpression exerts the opposite effects [30]. PAX3 also directly binds the p53 promoter to transcriptionally repress p53, regulating GSC proliferation and differentiation [31]. This established regulatory relationship in other stem cells provides a strong rationale to investigate whether PAX3 similarly influences the p53/p21 pathway during hDPSC senescence.

Building on these findings, we hypothesized that PAX3 may regulate hDPSCs proliferation, differentiation, and senescence. To test this, we silenced or overexpressed PAX3 in hDPSCs and assessed senescence-associated markers (p53, p21, and SA-β-gal), proliferation (CCK-8 assay), and osteogenic differentiation. Silencing PAX3 was associated with upregulation of p53 and p21 mRNA, increased SA-β-gal positivity, and reduced proliferation and osteogenic capacity, while PAX3 overexpression showed the opposite associations. These correlative findings are consistent with a potential role for PAX3 in hDPSC senescence, possibly involving the p53/p21 pathway. Nevertheless, alternative mechanistic interpretations exist. PAX3 has been reported to regulate other signaling pathways relevant to cellular senescence, including PI3K/Akt [8] and TGF-β/Smad [11], and the observed changes in p53/p21 expression may represent secondary consequences of PAX3-mediated effects on cell proliferation or stress responses rather than direct transcriptional regulation. Given that our data are limited to mRNA levels and cellular staining, direct causality cannot be established, nor can a specific mechanism through the p53/p21 pathway be confirmed at the protein level. Thus, while PAX3 correlates with the senescent phenotype, further investigations incorporating protein quantification, pathway-specific inhibition, and rescue experiments are required to delineate the underlying mechanism.

## Limitations

This study was conducted exclusively at the in vitro cellular level, and thus may not fully recapitulate the molecular mechanisms of hDPSCs senescence in vivo. The regulatory effect of PAX3 on the p53/p21 pathway was primarily assessed at the mRNA level using RT-qPCR, and protein-level validation via Western Blot was not performed in the current study. Furthermore, osteogenic differentiation was assessed primarily by ALP and alizarin red staining with semi-quantitative nodule counting, which limits the depth of functional characterization. Therefore, the proposed involvement of the p53/p21 pathway remains correlative and inferential, and the conclusions regarding the molecular mechanism should be interpreted with caution. Nevertheless, the observed correlation between PAX3 and p53/p21 expression aligns with the known transcriptional regulatory functions of PAX3, providing a reasonable basis for future mechanistic investigations. Future studies should include protein-level analyses, chromatin immunoprecipitation, promoter-reporter assays, and explore in vivo models and downstream signaling cascades to validate these findings and elucidate the mechanistic details.

## Data Availability

Data are available from the corresponding author on reasonable request (Dr. Wenjing Li, Email: [27801072@hebmu.edu.cn](mailto:27801072@hebmu.edu.cn) ).
